# Cervical intraepithelial neoplasia in women with transformation zone type 3: cervical biopsy versus large loop excision

**DOI:** 10.1111/1471-0528.17200

**Published:** 2022-05-26

**Authors:** Line Winther Gustafson, Anne Hammer, Mary Holten Bennetsen, Christina Blach Kristensen, Huda Majeed, Lone Kjeld Petersen, Berit Andersen, Pinar Bor

**Affiliations:** ^1^ Department of Public Health Programmes, Randers Regional Hospital University Research Clinic for Cancer Screening Randers Denmark; ^2^ Department of Clinical Medicine Aarhus University Herning Denmark; ^3^ Department of Obstetrics and Gynaecology Gødstrup Hospital Herning Denmark; ^4^ Department of Pathology Randers Regional Hospital Randers Denmark; ^5^ Department of Obstetrics and Gynaecology Horsens Regional Hospital Horsens Denmark; ^6^ Department of Obstetrics and Gynaecology Viborg Regional Hospital Viborg Denmark; ^7^ Department of Obstetrics and Gynaecology Odense University Hospital Odense Denmark; ^8^ Open Patient data Explorative Network, Department of Clinical Research University of Southern Denmark Odense Denmark; ^9^ Department of Obstetrics and Gynaecology Randers Regional Hospital Randers Denmark

**Keywords:** biopsies, cervical intraepithelial neoplasia, colposcopy, human papillomavirus, LLETZ, postmenopausal, transformation zone type 3

## Abstract

**Objective:**

To compare the proportion of cervical intraepithelial neoplasia grade 2 or higher (CIN2+) in cervical biopsies with that in large loop excision of the transformation zone (LLETZ) specimens in women aged ≥45 years with transformation zone type 3 (TZ3).

**Design:**

Multicentre cross‐sectional study.

**Setting:**

Three colposcopy clinics in the Central Denmark Region.

**Population:**

Women aged ≥45 years referred to colposcopy as a result of a positive human papillomavirus (HPV) test and/or abnormal cytology and with TZ3 at colposcopy.

**Methods:**

Women had multiple biopsies taken and an LLETZ was performed.

**Main outcome measures:**

Histologically confirmed CIN2+ in biopsies compared with that in LLETZ specimens.

**Results:**

Of 166 eligible women at colposcopy, 102 women with paired data from biopsies and LLETZ specimens were included for final analysis. The median age was 67.7 years (IQR 62.6–70.4 years), and most were postmenopausal (94.1%) and had undergone HPV‐based screening (81.3%). The CIN2+ detection rate was significantly higher in LLETZ specimens than in biopsies (32.4% vs 14.7%, difference 17.7%, 95% CI 6.3–29.0%), resulting in more than half of CIN2+ cases being missed in biopsies (54.5%, 95% CI 36.4–71.9%). The overall agreement between biopsies and LLETZ was 82.4% (95% CI 73.6–89.2%).

**Conclusions:**

CIN2+ detection is underestimated in women aged ≥45 years with TZ3 if detection relies on the results of biopsies alone. To reduce the risk of underdiagnosis and overtreatment, future studies should explore the use of new biomarkers for risk stratification to improve discrimination between women at increased risk of CIN2+ who need to undergo LLETZ and women who may undergo follow‐up.

## Abstract 2

**Linked article:** This article is commented on by Sarah Feldman, pp. 2141 in this issue. To view this mini commentary visit https://doi.org/10.1111/1471‐0528.17260

## 1 INTRODUCTION

Cervical cancer is the fourth most common cancer in women worldwide, with approximately 570 000 cases yearly and 311 000 deaths in 2018.^1^ In Denmark, cervical cancer is diagnosed in nearly 400 women each year, with approximately 100 deaths annually.^2^ Recent studies have reported higher incidence and mortality rates in older women than in younger women.^3^, ^4^ Moreover, older women are more commonly diagnosed with advanced‐stage disease and, hence, have a poorer prognosis.^5^ This may be because of insufficient screening, screening failure, diagnostic challenges and insufficient follow‐up of older women.

In postmenopausal women, the transformation zone (TZ) is often retracted into the cervical canal as a result of age‐dependent changes. Combined with epithelial atrophy, these factors challenge the complete visualization of the squamous–columnar junction (i.e. TZ type 3), including a potential precancerous lesion. These issues hamper the collection of targeted biopsies and increase the risk of missing disease located in the cervical canal. Histopathological examination of colposcopy‐directed biopsies is used for diagnosis, and several studies indicate that collecting multiple biopsies improves the chances for detection of cervical intraepithelial neoplasia grade 2 or higher (CIN2+).^6^, ^7^, ^8^ However, in women with TZ3, the collection of biopsies is likely to be compromised, resulting in an increased risk of underdiagnosis. Consequently, several guidelines suggest the use of large loop excision of the transformation zone (LLETZ) in women referred with abnormal cytology and TZ3 at colposcopy, to ensure correct diagnosis.^9^, ^10^, ^11^, ^12^ In women aged ≥45 years with TZ3, a diagnostic LLETZ may be offered to women either immediately or after insufficient sampling of histological material. However, performing an LLETZ on all women referred to colposcopy is likely to increase the risk of overtreatment and complications such as bleeding and stenosis, and stenosis could hamper subsequent follow‐up.^13^, ^14^ Our understanding of the magnitude of potential underdiagnosis when only taking biopsies and overtreatment following diagnostic LLETZ is lacking.

Therefore, we aimed to compare the CIN2+ detection rate in cervical biopsies with that in LLETZ specimens in women aged ≥45 years with TZ3.

## 2 METHODS

### 2.1 Study design and setting

This multicentre, cross‐sectional study was conducted from March 2019 through June 2021 at the Departments of Obstetrics and Gynaecology in the Central Denmark Region, Denmark (i.e. the Regional Hospital of Randers, Viborg and Horsens).

In Denmark, cervical cancer screening is offered to women aged 23–64 years. All procedures related to screening, diagnostic, follow‐up and treatment are free of charge for all citizens. Women aged 23–59 years are invited for cytology‐based screening every third year (age 23–49 years) or fifth year (age 50–59 years), whereas women aged 60–64 years are offered high‐risk human papilloma virus (HPV)‐based screening. Since January 2021, HPV‐based screening has been offered to women aged 30–59 years born on odd numbered dates, whereas women born on even numbered dates have continued with cytology‐based screening.^15^ Additionally, as a part of an interventional study in the Central Denmark Region that started in April 2019, women aged 65–69 years have been invited for one additional HPV screening test, with women testing positive for HPV undergoing cytology triage.^16^ Women participating in the present study were referred as summarised in Table S1.

### 2.2 Participants

Women were prospectively assessed for eligibility if they were aged ≥45 years, had an abnormal cervical cancer screening result (Table S1) and were referred to colposcopy. At colposcopy, women were considered eligible for inclusion if they had TZ type 3, according to the 2011 International Federation of Cervical Pathology and Colposcopy nomenclature.^17^ Women were excluded if they had previous excisional treatment or hysterectomy, received anticoagulant medical treatment, intended to get pregnant or underwent follow‐up for a previously diagnosed CIN. Moreover, women were excluded if LLETZ was not technically possible because of pain, a narrow vagina or severe atrophy of the vagina or cervix, making it difficult to distinguish the cervix from the vaginal wall.

All women received written and verbal information on the study, and upon inclusion the participating women signed an informed consent form.

### 2.3 Clinical management

Colposcopic examination of the cervix was performed after the application of acetic acid (3%) and biopsies were obtained with 3‐mm forceps from abnormal areas identified by colposcopy. If no abnormalities appeared, four blind biopsies were taken according to Danish national guidelines and the study protocol.^18^ At colposcopy, but prior to taking biopsies and performing the LLETZ, a liquid‐based cytology sample (SurePath™; BD, Franklin Lakes, NJ, USA) was collected using Cervex‐Brush® and/or EndoCervex‐Brush® (Rovers Medical Devices, Oss, the Netherlands). Finally, an LLETZ was performed immediately after colposcopy. The procedure was performed using local anaesthesia (Citanest Dental Octapressin®; Dentsply Sirona, York, PA, USA).

### 2.4 Data sources

From the medical records, the following data were obtained: colposcopy description, parity, medical history and daily medication. After colposcopy, women completed a questionnaire with questions on basic characteristics and behavioural risk factors. Information on previous cervical cancer screening results and results of study‐related cervical cytology, biopsies and LLETZ specimens were obtained from the nationwide Danish Pathology Databank, which stores information on all cyto‐ and histopathological examinations performed in Denmark since 1998 at an individual level.^19^

### 2.5 Cytology and histopathological examination

The Department of Pathology, Randers Regional Hospital, Denmark, is responsible for analysing all cervical cytology samples in the Central Denmark Region (approx. 90 000 samples annually). Cytology slides were interpreted by experienced cyto‐technicians using computer‐assisted microscopy (BD FocalPoint™ GS Imaging System). Results were categorised according to the Bethesda 2014 grading system.^20^ HPV DNA testing was performed using the cobas® 4800 system (Roche Diagnostic, Basel, Switzerland), which enables the individual detection of HPV16 and HPV18, and the pooled detection of 12 other high‐risk HPV types (31, 33, 35, 39, 45, 51, 52, 56, 58, 59, 66 and 68).^21^ Biopsies and LLETZ specimens were routinely examined at local pathology departments in Randers and Viborg. Histopathological outcomes were graded according to the cervical intraepithelial neoplasia (CIN) classification:^22^ normal, CIN1, CIN2, CIN3, unclassifiable CIN (i.e. the full height of the epithelium is not discernible), adenocarcinoma in situ (AIS) or cancer. Histopathological outcomes for biopsies and LLETZ were divided into two categories: <CIN2, defined as normal (including no dysplasia and inflammation) and CIN1; and CIN2+, defined as unclassifiable CIN, CIN2, CIN3 and cancer. The TZ was considered to be represented in biopsies if the specimen contained both squamous and glandular epithelium (on the surface or in a crypt). The LLETZ specimen was considered to be representative if the squamous–columnar junction (SCJ) was present on the surface of the tissue specimen.

### 2.6 Statistics

We used CIN2+ as the primary outcome, as this is the threshold for excisional treatment in women aged ≥45 years. CIN2+ detection in biopsies and LLETZ was reported using proportions and 95% confidence intervals (95% CIs), and McNemar’s χ^2^ (i.e. paired samples) was used for comparison of proportions across groups. The overall percentage agreement in histological diagnoses between paired biopsies and LLETZ was calculated and presented with 95% CIs. To test the robustness of the agreement calculation, we performed a sensitivity analysis excluding cases with unclassifiable CIN. Continuous variables were reported as medians and interquartile ranges (IQRs).

Data were entered and stored at Research Electronic Data Capture (REDCap, https://www.project‐redcap.org/).^23^, ^24^ STATA 16 (StataCorp LLC, College Station, TX, USA) was used for statistical analyses. A *p*‐value of <0.05 was considered statistically significant.

## 3 RESULTS

### 3.1 Study population

**FIGURE 1:**
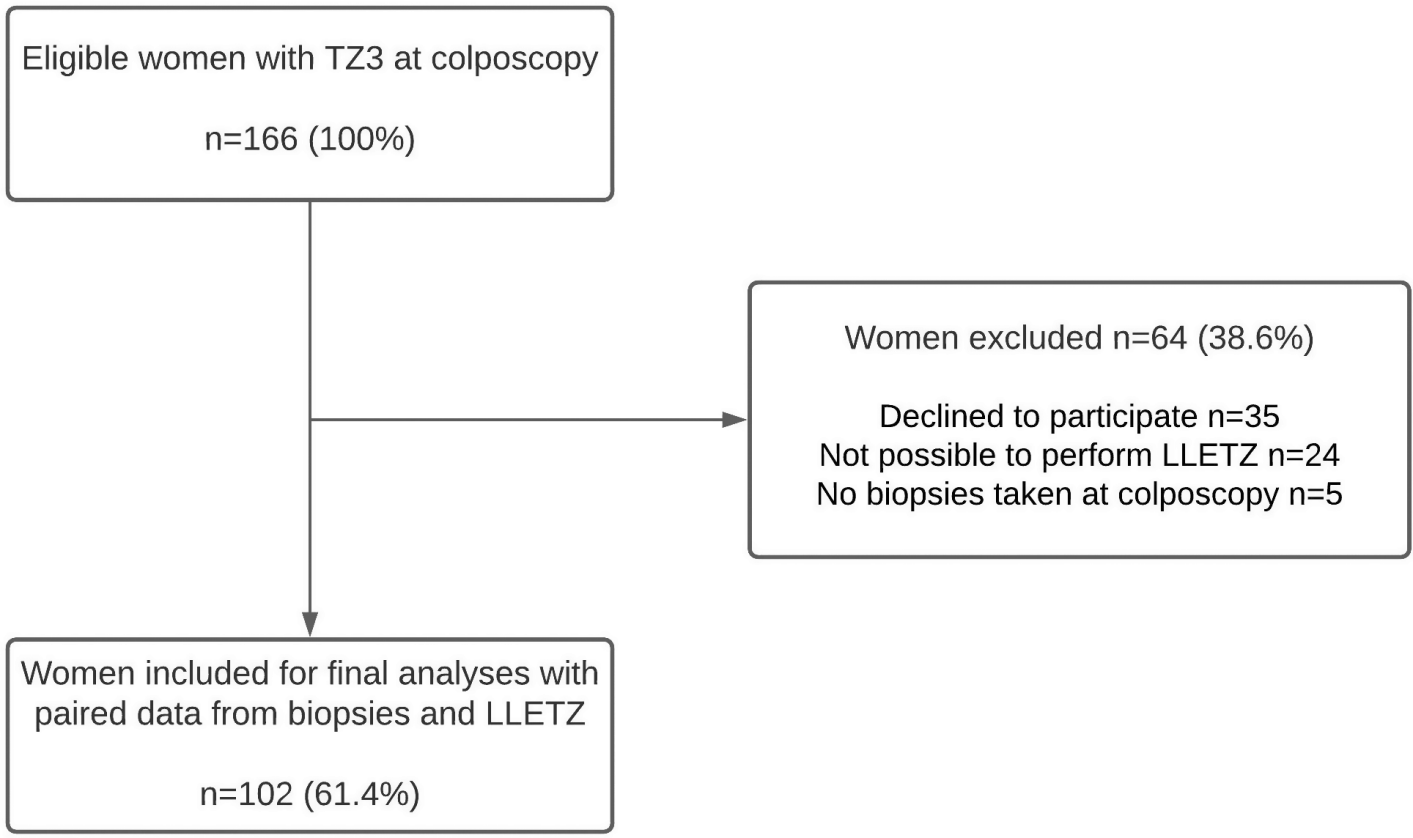
Flow chart of the study population

Of 166 women eligible at colposcopy, 35 (21.1%) declined to participate. For 24 women (14.5%), LLETZ was not technically possible (Figure 1), and another five women (3.0%) had no biopsies taken, leaving 102 women (61.4%) with paired samples for the final analyses (Figure 1). Although anticoagulant treatment was an exclusion criterion, three women receiving anticoagulant medical treatment were included by accident. The three women showed no excessive bleeding after LLETZ, and we decided to include these for the final analysis as they did not differ from other study participants with respect to basic characteristics.

The median age of the included women was 67.6 years (IQR 62.6–70.4 years), with the majority being postmenopausal (*n* = 96; 94.1%) (Table 1). Most women were non‐smokers (*n* = 72, 70.6%) and did not use vaginal hormone (*n* = 79, 77.5%). The majority had undergone primary HPV screening (*n* = 83, 81.3%), and 69 women (67.7%) had no previous record of atypical squamous cells of undetermined significance or worse (ASC‐US+). With regards to sexual behaviour, 58 women (56.9%) had had more than five lifetime sexual partners, and most women (82.4%) reported no new sexual partner within the past 2 years (Table 1).

**TABLE 1:** Basic characteristics of the women aged ≥45 years referred to colposcopy

	Women *n*, (%)(*N* = 102)
Median age (IQR)	67.6 (62.6–70.4)
Age groups (years)	
45–59	14 (13.7)
≥60	88 (86.3)
Body mass index (BMI)^a^	
Median BMI (IQR)	25.4 (21.8–28.2)
Current smoking^a^	
No	72 (70.6)
Yes	23 (22.6)
Parity^a^	
Nulliparous	8 (7.8)
Parous	90 (88.2)
Menopause status^a^	
Postmenopausal	96 (94.1)
Premenopausal	2 (2.0)
Current use of vaginal hormone^a^	
No	79 (77.5)
Yes	15 (14.7)
Use of hormone therapy during menopause^a^	
No	88 (86.3)
Yes	6 (5.9)
Referral status^b^	
Primary HPV screening	83 (81.3)
Primary cytology screening	19 (18.7)
Previous history of abnormal cytology (ASC‐US+)^b^	
No	69 (67.7)
Yes	33 (32.4)
Lifetime sexual partners^a^	
<5	31 (30.4)
5–10	36 (35.3)
>10	22 (21.6)
New sexual partners within the past 2 years^a^	
Yes	8 (7.8)
No	84 (82.4)
HPV vaccination^a^	
Yes	7 (6.9)
No	88 (86.3)

^a^ Self‐reported data.

^b^ Data on referred status and previous history of abnormal cytology was obtained from the Danish Pathology Databank. ASC‐US+ is defined as atypical squamous cells of undetermined significance or worse. The numbers and proportions vary because of missing data.

### 3.2 Colposcopy and histopathological outcomes

Fifteen women (14.0%) had abnormalities detected at colposcopy, with acetowhitening being the most common finding (73.3%). Five women (33.3%) had atypical vessels (data not shown). Most women (63.7%) had four biopsies taken, as recommended in the Danish guidelines; however, as a result of technical difficulties, 37 women (36.3%) had fewer than four biopsies taken (Table 2). The median depth of the LLETZ specimen was 10 mm (IQR 8–13 mm).

**TABLE 2:** Histopathological results detected in biopsies and LLETZ specimens

Variable	Biopsies	LLETZ
Histopathological result overall and grouped into <CIN2^a^ and CIN2+^b^		
	*n* %, (95% CI)	*n* %, (95% CI)
Normal	83 81.4, (72.4–88.4)	62 60.8, (50.6–70.3)
CIN1	4 3.9, (1.1–9.7)	7 6.9, (2.8–13.6)
CIN2	3 2.9, (0.6–8.4)	5 4.9, (1.6–11.1)
CIN3	4 3.9, (1.1–9.7)	17 16.7, (10.0–25.3)
Unclassifiable CIN	8 7.8, (3.4–14.9)	11 10.8, (5.5–18.5)
<CIN2	87 85.3, (76.9–91.5)	69 67.7, (57.7–76.6)
CIN2+	15 14.7, (8.5–23.1)	33 32.4, (23.4–42.3)
Total	102 (100.0)	102 (100.0)

Representation of squamous and endocervical epithelium in the specimen		
	*n* %, (95% CI)	*n* %, (95% CI)
<CIN2		
Yes	48 55.2, (44.1–65.9)	64 92.8, (83.9–97.6)
No	39 44.8, (34.1–55.9)	5 7.2, (2.4–16.1)
CIN2+		
Yes	12 80.0, (51.9–95.7)	33 100, (89.4–100.0)
No	3 20.0, (4.3–48.1)	0

CIN2+ result stratified by referral status^c^		
	*n* %, (95% CI)	*n* %, (95% CI)
HPV+ and normal cytology		
<CIN2	43 42.2, (32.4–52.3)	42 41.2, (31.5–51.4)
CIN2+	<3	<3
HPV+ and ASC‐US+		
<CIN2	28 27.5, (19.1–37.2)	13 12.7, (7.0–20.8)
CIN2+	8 7.8, (3.4–14.9)	23 22.5, (14.9–31.9)
Cytology‐based screening		
<CIN2	13 12.7, (7.0–20.8)	11 10.8, (5.5–18.5)
CIN2+	6 5.9, (2.2–12.4)	8 7.8, (3.4–14.9)

Histopathological result statisfied by the number of biopsies taken at colposcopy				
	*n* %, (95% CI)		*n* %, (95% CI)	
	Biopsies <4	Biopsies (≥4 biopsies)	Biopsies <4	Biopsies (≥4 biopsies)
<CIN2	33 32.4, (23.4–42.3)	54 52.9, (42.8–62.9)	23 22.5, (14.9–31.9)	46 45.1, (35.2–55.3)
CIN2+	4 3.9, (1.1–9.7)	11 10.8, (5.5–18.5)	14 13.7, (7.7–22.0)	19 18.6, (11.6–27.6)
Total	37 36.3, (27.0–46.4)	65 63.7, (53.6–73.0)	37 36.3, (27.0–46.4)	65 63.7, (53.6–73.0)

Abbreviation: CIN, cervical intraepithelial lesions.

^a^ <CIN2 is defined as: normal or CIN1.

^b^ CIN2+ is defined as: CIN unclassifiable, CIN grades 2 and 3, and cancer. LLETZ: large loop excision of the transformation zone.

^c^ Women who underwent HPV‐based screening without reflex cytology are excluded (*n* = 3). HPV+: Any high‐risk HPV type.

Histopathological examination of biopsies showed that 87 women (85.3%, 95% CI 76.9–91.5%) had <CIN2, and 15 women (14.7%, 95% CI 8.5–23.1%) had CIN2+ detected (Table 2). Histopathological examination of the LLETZ specimens revealed that 69 women (67.7%, 95% CI 57.7–76.6) had <CIN2, which was statistically lower compared to the biopsies (85.3% vs 67.7%, *p* < 0.01). Similarly, the CIN2+ detection was significantly higher in the LLETZ specimens than in the biopsies (32.4% vs 14.7%, *p* < 0.01) (Table 2), corresponding to 54.5% of CIN2+ cases being missed in biopsies (95% CI 36.4–71.9) (Table 3). The overall percentage agreement between biopsies and LLETZ specimens was 82.4% (95% CI 73.6–89.2%) (Table 3). Excluding unclassifiable CIN from the analysis did not change the percentage agreement (82.4% vs 86.2%, *p* = 0.47).

**TABLE 3:** Agreement in histopathological results between biopsies and LLETZ

Cervical biopsy result	LLETZ result			
		<CIN2^a^	CIN2+^b^	Total
	<CIN2^a^	69 (67.6%)	18 (17.6%)	87 (85.3%)
	CIN2+^b^	0	15 (14.7%)	15 (14.7%)
	Total	69 (67.6%)	33 (32.4%)	102 (100%)
	Agreement		84/102 = 82.4% (95% CI 73.6–89.2%)	
	Biopsy underestimates disease in all women		18/102 = 17.6% (95% CI 10.8–26.4%)	
	Biopsy underestimates disease in women with CIN2+ detected		18/33 = 54.5% (95% CI 36.4–71.9%)	
	Biopsy overestimates or removes disease		0/102 (0%)	

Abbreviation: CIN, cervical intraepithelial lesion.

^a^ <CIN2 is defined as: normal or CIN1.

^b^ CIN2+ is defined as: CIN unclassifiable, CIN grades 2 and 3, and cancer.

Of the 18 women with no evidence of CIN2+ in biopsies but with CIN2+ detected in the LLETZ specimen, 12 women (66.7%) had the TZ represented in the biopsies. All 18 women with CIN2+ detected by LLETZ had the SCJ included in the LLETZ specimen (Table 2). Of these 18 women, 16 (88.9%) were referred with a positive HPV test (81.3% non‐16/18‐HPV) and ASC‐US+ in their reflex cytology (data not shown).

With respect to the referral status of the women and risk of CIN2+, we found that women testing positive for HPV with ASC‐US+ on reflex cytology had an increased risk of being diagnosed with CIN2+, as compared with women testing positive for HPV with normal reflex cytology (Table 2). The positive predictive value of ASC‐US+ on reflex cytology in women testing positive for HPV (*n* = 80) was 63.9% (95% CI 46.2–79.2%).

A total of 33 women (32.4%) had a previous abnormal screening history, of which 23 (22.5%) and 10 (9.8%) women were diagnosed with <CIN2 and CIN2+ detected in the LLETZ specimen, respectively. In women with a normal screening history, 46 women (45.1%) had <CIN2 and 23 women (22.5%) had CIN2+. In women with previous abnormal cytology (*n* = 33, 32.4%), 24 women (72.7%) had previously noted low‐grade changes and nine women (27.3%) had previously noted high‐grade changes (data not tabulated). Moreover, in women with colposcopic abnormalities (*n* = 15), six (40.0%) had <CIN2 and nine (60.0%) had CIN2+ in their LLETZ specimens (data not tabulated).

The results of women with the representation of the TZ in biopsies and the SCJ in the LLETZ specimen are summarised in Table 2. Of note, in women with representation of the TZ in at least one of the biopsies, the biopsies missed CIN2+ lesions in 21 out of 33 (63.6%, 95% CI 45.1–79.6%) cases detected in the LLETZ specimens (Table 2).

## 4 DISCUSSION

### 4.1 Main findings

In this multicentre, cross‐sectional study on women aged ≥45 years with TZ3, we found that biopsies underestimated over half of the CIN2+ cases detected in the LLETZ specimens. Relying on the results of biopsies in women with TZ3 carries a risk of delaying diagnosis and treatment, as the TZ was significantly less represented in biopsies compared with LLETZ specimens. However, as 67.7% of women in the present study had <CIN2 detected in the LEETZ specimen, our study also suggests a profound risk of overtreatment. Thus, weighing the risk of underdiagnosis against overtreatment is important in the clinical management of women with TZ3 at colposcopy.

### 4.2 Strengths and limitations

A major strength of this multicentre study was the use of paired samples (i.e. multiple biopsies and LLETZ specimens), which minimised the risk of confounding. The majority (93.9%) of biopsies and LLETZ specimens were analysed at the same pathology department in Randers, limiting inter‐laboratory variation. The present study contributes results from a unique population of women aged ≥45 years attending colposcopy. Our results may be generalisable to other populations of women with TZ3 and with a comparable cervical cancer incidence who have undergone a similar screening and triage algorithm.

A number of limitations should also be addressed. The sample size was rather small, which makes the results less robust. We did not use an external reference reviewer for histopathological examination, which could increase the risk of inter‐ and intra‐observer variation. The pathologists were aware of the histopathological result of the biopsies before the LLETZ specimens were assessed. However, this reflects real‐life practice and despite this knowledge half of the biopsies were still evaluated as normal. We cannot rule out that some women may have been misclassified as having a TZ3 instead of TZ2. However, as the outcome (CIN2+) is not affected by the type of TZ, the risk of bias is considered minimal, but potential misclassification might explain the high number of biopsies with the TZ being represented. We cannot rule out that selection bias might have occurred when women were booked for colposcopy; however, we consider selection bias to be minimal as women were enrolled consecutively. We acknowledge that results based on multiple biopsies may not be generalisable to other countries where fewer biopsies are taken. As a result, implementation of diagnostic LLETZ in such settings may be even more efficient than in our setting. In our study, some women (aged 65–69 years) participated in a continuing trial offering them an additional HPV test.^16^ These women could have been HPV‐positive for several years, which could have contributed to the high CIN2+ detection rate found in our study.

### 4.3 Interpretation

The CIN2+ detection rate in this multicentre study is somewhat higher than detection rates reported in other studies investigating women aged ≥55 years (32% vs 15–25%).^25^, ^26^, ^27^, ^28^, ^29^ A recent study including women (aged ≥70 years) undergoing HPV‐based screening reported that 18% of women who had histological sampling performed (514 of 2782) had CIN2+ detected.^25^ Taking the age into consideration, we would expect these women to have a TZ3 at colposcopy. Our findings suggest that the CIN2+ detection rate in the previous study may have been underestimated, as not all women had biopsies collected and the histopathological outcome was based on the combined results of biopsy and LLETZ. Aarnio et al. explored risk of CIN2+ among women (*n* = 40, mean age 58 years) with persistent HPV infection and normal cytology by collecting biopsies and performing an LLETZ at the same visit.^28^ They reported CIN2+ in 15% (6/40) of women with LLETZ, whereas biopsies failed to detect any of these CIN2+ cases. Another study on women who were HPV‐positive/cytology‐normal (mean age 59.5 years) reported CIN2+ in 25% (6/24) in a subgroup of women who were offered diagnostic conisation as a result of TZ3.^27^ The higher CIN2+ detection rate in our study compared with previous studies may be explained by differences in clinical management and the characteristics of study participants across studies, such as the inclusion of women testing positive for HPV with abnormal cytology in the present study, unlike previous studies.

The current Danish National Guidelines state that four biopsies should be taken at colposcopy regardless of a visible lesion and referral status.^18^ These recommendations are based on results from studies showing that collecting multiple biopsies increases the possibility of detecting CIN2+.^6^, ^7^, ^8^ In women with a visible TZ, biopsies may be used as the reference standard instead of LLETZ. Thus, a recent study reported a 95% concordance in detecting CIN2+ between representative biopsies and LLETZ in women with a visible TZ.^8^ However, despite taking multiple biopsies in our study, more than 50% of CIN2+ lesions were missed. In women who had biopsies with the TZ being represented, 63.6% of CIN2+ cases diagnosed in the LLETZ specimen were missed. Caution is therefore advised when interpreting non‐representative biopsies from women with TZ3. To improve the visualization of the TZ and a potential lesion, a few trials have suggested pre‐colposcopic treatment with estrogen or misoprostol.^30^, ^31^, ^32^ Unfortunately, these studies suffer from a small sample size, and future well‐conducted clinical trials are therefore needed to clarify the potential impact of this treatment, including the appropriate dose and duration of treatment. Endocervical curettage (ECC) is another possibility for obtaining histopathological material in women with TZ3. Studies have indicated that the use of ECC may increase the diagnostic yield of CIN2+, particularly in older women.^33^, ^34^ However, the use of ECC remains controversial as ECC has a higher rate of unsatisfactory histopathological results than biopsies and may be more painful for the women. For these reasons, ECC is not routinely recommended in Denmark.^18^

Cervical cancer screening and the diagnostic work‐up of women screening positive is a delicate balance between benefits, in terms of preventing cancer cases, and harms, in terms of complications related to preventive treatment. The choice of management strategy should preferably be based on the result of the referral test, screening history, the women’s preferences and other risk factors. Offering LLETZ to all women testing positive for HPV, regardless of triage and risk profile, would lead to overtreatment and possible complications. This statement is supported by our study data showing that more than half of the women (60.8%) had a normal histopathological result in their LLETZ specimen. This estimate of overtreatment corresponded to the 50–75% with normal histopathological results reported in the above‐mentioned studies.^26^, ^28^, ^29^ Although women aged ≥60 years have been shown to prefer the adverse effects of overtreatment over the risk of underdiagnosis,^35^ additional studies are needed to better identify women at increased risk of CIN2+, while allowing women at lower risk to undergo follow‐up. p16/Ki67 dual stain could be a potential risk marker as studies have demonstrated that it has a higher sensitivity and negative predictive value for detecting CIN2+ than cytology in a screening population of younger women.^36^, ^37^ However, not much is known about the clinical utility of the p16/Ki67 dual stain in a referral population of women aged ≥45 years. Likewise, studies on methylation markers as a triage strategy for women testing positive for HPV have shown promising results.^38^ Exploring whether these or other biomarkers could enable a reliable risk stratification of women with TZ3 is of utmost clinical importance to reduce the risk of overtreatment while simultaneously securing adequate diagnosis. Until these biomarkers are properly validated for use as risk markers in a similar population, our results suggest that women testing positive for HPV with normal cytology could undergo follow‐up, whereas women testing positive for HPV with ASC‐US+ in their reflex cytology could undergo LLETZ.

## 5 CONCLUSION

Our findings indicate that CIN2+ detection in women aged ≥45 years with TZ3 is underestimated when detection relies solely on the results of biopsies, compared with LLETZ specimens. CIN2+ lesions are even missed in women with both endocervical and squamous cells represented in the biopsies. However, performing LLETZ for all women with TZ3 at colposcopy is likely to raise the risk of overtreatment significantly. To individualise diagnostic work‐up and treatment, and to minimise the risk of underdiagnosis and overtreatment, future studies should explore the use of new biomarkers for individual risk stratification.

### CONFLICT OF INTERESTS

LWG and LKP have received speaker’s fees from Astra Zeneca outside of the submitted work. LWG and BA are participating in other studies with HPV test kits and CINtec® PLUS kits sponsored by Roche. AH reports receiving reagents from Roche Denmark at a reduced cost, outside of the submitted work. MHB, CBK, HM and PB report no conflicts of interest. Completed disclosure of interests form available to view online as supporting information.

### AUTHOR CONTRIBUTIONS

Conceptualisation and study design: LWG, AH, BA, PB and LKP. Recruitment, data collection, data management and analysis of samples: CBK, MHB, HM, PB and LWG. Data analyses: PB, AH, LWG, BA and LKP. LWG prepared the first draft with guidance/supervision from BA, LKP, PB and AH. Critical revision of the article for important intellectual content was provided by CBK, HM, MHB, LWG, BA, PB, AH and LKP. All authors approved the final version for publication.

### DETAILS OF ETHICS APPROVAL

This study was listed at the record of processing activities for research projects in the Central Denmark Region (J.no: 1–16–02‐528‐18). The Central Denmark Region Committees on Health Research Ethics decided that according to the Consolidation Act on Research Ethics Review of Health Research Projects, Consolidation Act number 1083 of 15 September 2017, section 2 (1), this study was not notifiable to the committee (J.no.: 1–10–72‐4‐17).

## Supporting information


Table S1

Appendix S1

Appendix S2

Appendix S3

Appendix S4

Appendix S5

Appendix S6

Appendix S7

Appendix S8

## Data Availability

The data that support the findings of this study are available on request from the corresponding author. The data are not publicly available due to privacy or ethical restrictions. Data can be made available on request from researchers who meet the criteria for access to patient's confidential data and upon approval from the Danish Data Protection Agency.
