# Supplementary material for: Cervical intraepithelial neoplasia in women with transformation zone type 3: cervical biopsy versus large loop excision
Source: BJOG. 2022 May 26;129(13):2132–40. doi: 10.1111/1471-0528.17200 (PMC9796102; doi:10.1111/1471-0528.17200)
Supplement: Supplementary file 1 — Table S1 [file BJO-129-2132-s002.docx]

**Supporting Information.**

**Table S1:** Management of women after an abnormal screening result according to the Danish cervical cancer screening

| **Result of primary screening method** | | **Reflex testing** | **Management** |
| --- | --- | --- | --- |
| **Women aged 30-59 who undergo cytology-based screening** | | | |
|  | ASC-US | Positive reflex HPV test | Direct referral to colposcopy |
|  |  | Negative reflex HPV test | Return to screening program |
|  | LSIL | Reflex HPV testing not recommended | Repeat cytology test after six months |
|  | ASC-H, AGC, AIS, or HSIL | Reflex HPV testing not recommended | Direct referral to colposcopy |
| **Women aged 30-59 who undergo HPV-based screening** | | | |
|  | HPV positive | ASC-US, LSIL on cytology triage and additional triage positive* | Direct referral to colposcopy |
|  |  | ASC-US, LSIL on cytology triage and additional triage negative* | Repeat HPV testing after one year |
|  |  | AGC, ASC-H, HSIL, AIS on cytology triage | Direct referral to colposcopy |
|  |  | Normal cytology on cytology triage | Repeat HPV testing after one year |
| **Women aged 60-64 who undergo HPV-based screening** | | | |
|  | Positive for HPV 16 or 18** | Not required | Direct referral to colposcopy |
|  | Positive for other HPV types | ASC-US or worse on reflex cytology | Direct referral to colposcopy |
|  |  | Normal on reflex cytology | Repeat HPV test after one year*** |

Abbreviations: Women aged ASC-US: atypical squamous cells of undetermined significance, LSIL: low-grade squamous intraepithelial lesion, HSIL: High-grade squamous intraepithelial lesion, Atypical squamous cells-cannot exclude HSIL (ASC-H), Atypical glandular cells (AGC), Adenocarcinoma in situ (AIS), HPV: human papilloma virus. *Additional triage from January 2021 (type of triage dependent on region). In Central Denmark Region additionally triage is p16/Ki67 dual stain. ** Including other high risk (hr) HPV types in combination with 16 and/ or 18. *** Two positive tests with persistent other hrHPV types will lead to referral to colposcopy from January 2021. Before that date women were referred using ASC-US+ as threshold.
